# Social preferences for ecosystem services in a biodiversity hotspot in South America

**DOI:** 10.1371/journal.pone.0215715

**Published:** 2019-04-22

**Authors:** Iñigo Bidegain, Claudia Cerda, Emilia Catalán, Antonio Tironi, César López-Santiago

**Affiliations:** 1 Faculty of Forest Sciences and Conservation of Nature, Universidad de Chile, Santiago, Chile; 2 Department of Ecology, Social-Ecological Systems Laboratory, Universidad Autónoma de Madrid, Madrid, Spain; 3 Cooperativa de Trabajo Liken Ltda., Santiago, Chile; 4 Cienciambiental Consultores S.A., Santiago, Chile; 5 Fundación CTF., Santiago, Chile; Centre for Cellular and Molecular Biology, INDIA

## Abstract

Identifying which ecosystem services are relevant to different stakeholders and understanding stakeholders’ perceptions of such services is useful for making informed decisions, especially in regions of the world where the achievement of biodiversity conservation goals is threatened by economically productive activities. In this article, we assess social preferences for ecosystem services in a biodiversity hotspot in central Chile. We use a consultative case study to ask local stakeholders (n = 70) from the Campana Peñuelas Biosphere Reserve to identify the most important ecosystem services the area provides for them and inquire about the perceived vulnerability of the services to changes in the future. We also explore the association between the perceived importance of ecosystem services and the sociodemographic and cultural characteristics of the respondents, which allows us to identify contrasting stakeholder perceptions of different ecosystem services. The most important services for local actors were the drinking water, fresh air and climate change control, genetic pool of plant communities in central Chile, and educational value. From the perspective of local actors, the services that could be threatened by negative changes in the future in terms of their provision included the possibilities of developing conservation activities focused on iconic threatened animal and plant species, water regulation, food from agriculture, and drinking water. Contrasting perceptions about the importance of ecosystem services emerged among stakeholders. While small farmers and members of local organizations attributed higher importance values to provisioning services, scientists and rangers and administrators of protected areas as well as teachers, NGO members and local government employees attributed more importance to the regulating and cultural services associated with threatened species. Our results can serve as a source of information for the planning and decision-making processes related to the search for socially and ecologically sustainable solutions for land use management.

## Introduction

In recent decades, the concept of ecosystem services (ES) has had important impacts in both scientific and political forums [1,2]. ES can be defined as the aspects of ecosystems that are used (either actively or passively) to maintain human well-being [3]. This definition considers ecosystem organization, processes and functions utilized by humanity [3]. Policy initiatives, such as the Aichi Targets and the Intergovernmental Platform on Biodiversity and Ecosystem Services (IPBES) that explicitly recognize the importance of the ecosystem services approach to ecosystem management have stimulated assessments and valuations of ecosystem services in different regions of the world [4]. Although these policy platforms have explicitly recognized the importance of understanding the social dimensions of ecosystem services (e.g., assessing societal preferences for and perceptions of ecosystem services using non-economic approaches [5], uncovering divergent interests regarding ecosystem services from different local actors [6,7], or understanding how people respond to the management of such services [5], the assessment of these social dimensions of ecosystem services is still lacking. Much work has been done to conduct ecological assessments and economic valuations of ecosystem services [6,8]. The ecosystem services approach recognizes that healthy ecosystems depend not only on the ecological properties of ecosystems but also on their capacities to fulfil social needs [9]. In addition, the economic valuation of ecosystem services (i.e., the process of valuing the contributions of the ecosystem services and biodiversity at the level of the life and well-being of social actors, conceived in terms of individual utility [10] does not capture the full range of benefits people obtain from ecosystems [11,12]. If only ecological and economic criteria are considered in the assessments of ecosystem services, it can lead to conflicts in ecosystem management when social contexts are not appropriately recognized [13,14]. In this regard, identifying which services are relevant to different stakeholders and understanding stakeholders’ perceptions of such services [6,15–18] is relevant to making informed decisions, especially in regions of the world where the achievement of conservation goals is threatened by economically productive activities. On the one hand, ecosystem management is largely about regulating human actions towards ecosystem services [5]. Human actions are conditioned by the perceived benefits that people get from ecosystems and consequently such perceptions of benefits affect engagements or not in behaviours that ensure the continuous flow of desired ecosystem services ([5]: 181). On the other hand, different stakeholders can have different relationships with the same ecosystem. For example, scientists and administrators of protected areas may value a natural area because they want to safeguard threatened species and they recognize the scientific and educational value of the ecosystem. Local farmers can value the same area because their lifestyles are based on agricultural and farming activities; additionally, they may be guided by traditional ecological knowledge. Furthermore, tourists and urban-dwellers may value the area because they can appreciate its scenic beauty, but they do not have a close link or a long-standing connection to the ecosystem [6,7]. Divergent social interests may lead to conflicts over the use of territory, which threatens the achievement of conservation goals [19], and these conflicts can lead to the development of policies that result in very different outcomes and involve different beneficiaries [20]. Including *a priori* analyses of the social dimension of ecosystem services as part of ecosystem management may contribute to improving the provision of ecosystem services for all stakeholders, thus reducing conflict [8].

In this article, we assess social preferences for ecosystem services in one of the most important regions of the planet regarding the conservation of biodiversity, i.e., central Chile [21]. We use a consultative case study and ask local stakeholders from the Campana Peñuelas Biosphere Reserve about their preferences for different ecosystem services that flow in the area. Local stakeholders are the local government, enterprise managers/owners, small farmers, representatives of local organizations, and tourism workers. We also included educators from schools and colleges and scientists working on conservation and environmental topics in the area.

Specifically, we a) analyse the relative importance and the perceived vulnerability that the different stakeholders attribute to different ecosystem services, and b) explore the association between the perceived importance of ecosystem services with the sociodemographic and cultural characteristics of the respondents to identify contrasting perceptions of stakeholders regarding different ecosystem services.

Our study is framed by the need to strengthen the evidence of the links between natural systems and local communities in the context of biosphere reserve management in Chile [22]. For example, the current methodology for management planning in the Chilean System of Protected Areas of the State [23], which encompasses several protected areas that are part of biosphere reserves, involves not only ecological criteria but also human-well-being. Such a demand requires a process for implementing different approaches to garner local participation [24], where the assessment of preferences for ecosystem services can represent the beginning of a comprehensive understanding of the complex relationships between humans and natural systems [6,7]. Understanding how people use and value ecosystems is fundamental to achieving effective and equitable conservation, and the type of valuation presented here can help provide this information [5].

Given the scarce information on different local stakeholder preferences for ecosystem services in the study area, our research study has a consultative participatory characteristic [25] and adopts a semi-structured interview approach. According to Pretty et al. [25], the consultative participation approach is considered very appropriate when social actors respond to questions about their perceptions and knowledge on a topic, such as in our case. In addition, a semi-structured approach can be useful when the objective is to characterize social actors according to their perceptions of ecosystem services and use this information to provide initial images of diverging interests among stakeholders for ecosystem services [6,7]. The information generated with our approach can contribute to the future design and implementation of stronger participative methods, such as in depth interviews [26] or deliberative participation [5,27], that benefit from questionnaire results on the perceptions of ecosystem services.

At a global scale, with our study, we contribute to the existing literature on the social dimension of ecosystem services (e.g., [7,15,28,29]) by providing a new assessment of social preferences for ecosystem services in a globally relevant biosphere reserve in South America. South America has been recognized as a region that urgently requires more research on how to effectively conserve ecosystems while incorporating human needs and values [30]. Furthermore, our study has local relevance. Given the distinctive characteristics of the relationships between humans and ecosystems, many regions of the world are attempting to implement policies that require data on particular ecosystems [31,32]. Societies and ecosystems around the world differ [32]; thus, local studies of ecosystem services are necessary if we want to achieve the objectives of biosphere reserves and find sustainable solutions that balance social and economic development with biodiversity conservation.

## Methods

### Study area

**Fig 1:**
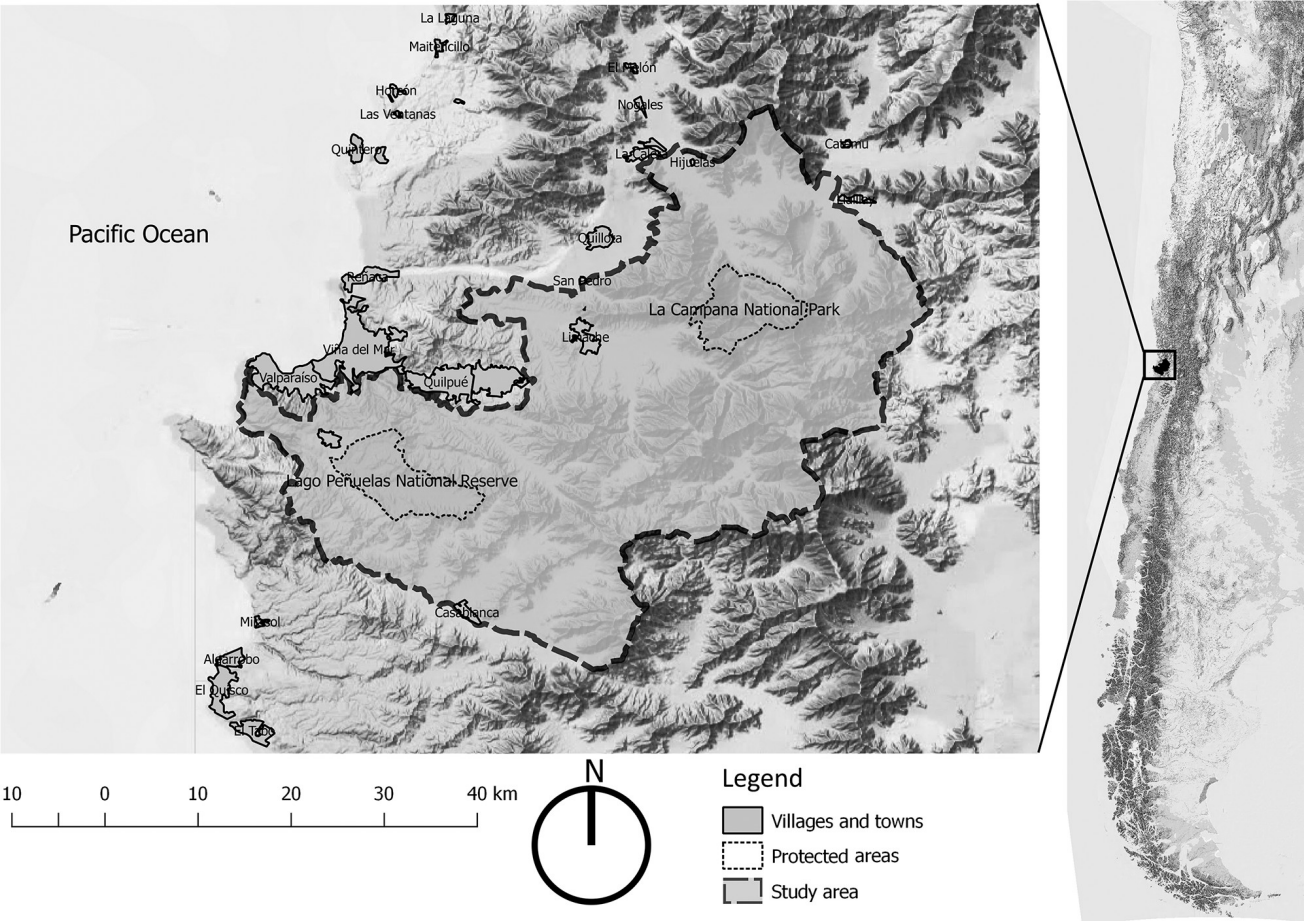
Location of the Campana Peñuelas biosphere reserve.

We conducted the study in a biosphere reserve in central Chile (Fig 1). This biosphere reserve was created in 1984 (with an area of 17.095 ha) and was expanded in 2009 (to an area of 238.216 ha).

The biosphere reserve is located between the two most populated regions of Chile, i.e., the Metropolitan (which contains the capital, Santiago) and Valparaíso, which together contain nearly half of the country’s population [21]. The main ecosystems of the biosphere reserve are Mediterranean sclerophyllous forests and scrublands, which together constitute a biodiversity hotspot [33]. The core areas are La Campana National Park and Lake Peñuelas National Reserve. The park (La Campana) is recognized as an international icon of biodiversity conservation in central Chile. The flora of the park consists of approximately 430 native species, of which more than half are endemic to Chile, and the abundant vegetation allows for the subsistence of a variety of species of fauna [34]. Lake Peñuelas National Reserve occupies an area of 9,260 ha and contains a permanent freshwater lake that supplies water to the cities of Valparaíso and Viña del Mar. The natural vegetation within the reserve is a mixture of sclerophyll forest and scrub, which forms an important centre of faunal and floral diversity and is an ecologically sensitive area [35]. However, the degree of environmental deterioration within the reserve is threatening its diversity [36,37]; for example, the forest fires, domestic livestock entering protected areas, and illegal extraction of Chilean palm (*Jubaea chilensis*) seeds are important threats [22]. The main human activities in the biosphere reserve are agricultural and silvicultural, but the area also hosts important environmental education programmes.

### Study design

#### Selection of respondents

We selected key local actors with different interests and activities within the territory of the biosphere reserve (Table 1). We define key local actors as those who have a strong connection to decision making in the area and/or those who have strong interests in local natural resources management; additionally, the key local actors live and/or work in the study area [38]. The objective of the sampling strategy was not to obtain a statistically significant number of surveys; rather, the goal was to obtain the maximum variety of opinions. As such, we were careful to incorporate actors from the different cultural groups present in the reserve in such a way as to obtain a diverse representation of the different points of view that determine the values in the local socio-ecosystem [22,38,39]. For the identification of actors we reviewed local literature (e.g., [40,41]) related to the study area that identified the primary activities that have developed in the area as well as the key actors related to those activities. In addition, we used the information on the distribution of predominant social groups in the biosphere reserve according to rural census localities [21]. To strengthen the information obtained by the literature review, we asked managers of protected areas to recommend key actors that could be interviewed. We began by asking managers of protected areas as they are more accessible for interviews. We asked them to identify actors who are in favour of conservation and actors whose activities seem to be in conflict with the conservation goals of the biosphere reserve. A list of potential key local actors was then compiled. We identified employees of the local government and enterprise managers/owners as key local actors with strong connections to decision making in the area. Additionally, we identified small farmers, representatives of local organizations, and tourism workers as key local actors with strong interests in local natural resources management. We also included a group of educators from schools and colleges as actors who could influence the long-term valuation of the local natural system. Scientists working on conservation and environmental topics in the area were also interviewed as they might influence decision-making processes.

**Table 1:** Stakeholders involved in the study and some of their characteristics.

Stakeholder	Description	Number of respondents
Scientists	Scientists are a group of environmental researchers with the highest levels of formal education; they specialize in ecology and agronomics. They all work at universities. They have a high level of ecological knowledge of the study area, but most live outside the area.	6
Employees of the Chilean National Forest Corporation (CONAF)	Public employees who correspond to park rangers; this category also includes administrators of the public protected areas located within the biosphere reserve. Both are interested in the conservation of biodiversity in the area.	7
Educators in schools and colleges	Teachers with a high level of formal education and a rural sense of place. They are not strictly related to environmental education or research, but they live in the study area.	6
Enterprise managers/owners	A heterogeneous group mainly composed of managers of medium-sized local companies; they are highly educated. These companies are mainly focused on agronomics, farming and real estate.	8
Employees of the local government	This group is composed of highly formally educated people who work for the public local administration. They do not have an environmental focus in their management and are closely linked to the study area.	7
NGO members	A group composed of locals with environmental concerns who actively participate in environmental or animal-related organizations.	10
Members of local organizations	Actors who actively participate in small organizations such as neighbourhood boards, foundations or indigenous communities. Such organizations are not focused on the conservation of biodiversity.	8
Small farmers	A group composed of local herders, farmers and beekeepers. These people have a strong rural sense of place, a low-to-medium level of formal education and a close link to the study area. Local herders and farmers represent a group to which environmental degradation practices are attributed.	12
Tourism workers	Managers of small-to-medium-sized local companies focused on tourism. Some are ecotourism companies, but they are not strictly related to environmental management or biodiversity conservation.	6
**Total**		**70**

Source: Adapted from Cerda and Bidegain [22].

Table 1 presents the stakeholders involved in the study and some of their characteristics.

Actors were contacted by the research team by email, telephone, and in person. The snow-ball technique was used with each contacted actor [38]; this technique consists of asking each interviewee to identify additional key people in the community that know the territory and its casuistry.

#### Design of the semi-structured interview

The first step in the interview design process was to identify the ecosystem services that flow in the area. For the identification of services, we followed other experiences in this topic of research (e.g., [42]). It has been suggested that one way to identify the ecosystem services to be valued is to consider the information available in the literature about the study area [42]. Thus, we conducted an extensive literature review of published scientific articles in different scientific data bases. We also reviewed national books (e.g., [21,34,37,43–45]) and book chapters (e.g., [46]). To strengthen the information search, technical documents (e.g., [47]) as well as student theses (e.g., [40,41]) were consulted. The extensive review of the scientific literature enabled the identification of some potential ecosystem services that flow in the area, and it enabled the validation of the list of potential key actors identified in the previous phase. However, with the exception of a few articles (e.g., [48,49]) most of the research addressed ecological questions (e.g., [50]), described the ecology, threats and management of the Chilean Palm (e.g., [51], or analysed disturbances of forest fires to the Chilean palm populations (e.g., [52]), revealing that there were few published scientific studies that explicitly addressed aspects of human dimensions, participation or ecosystem services. Although the studies by Cerda et al. [48] and Cerda and Losada [49] explored social perceptions about biodiversity and some benefits provided by protected areas, these studies only interviewed visitors of the protected areas present in the biosphere reserve. In this regard, the technical documents, books and student theses were more useful for identifying ecosystem services given that the human dimension of conservation was more frequently addressed in these types of literature, although without a specific focus on ecosystem services.

Forty-one potential ecosystem services to be included in the interview—all delivered by the ecosystems of the biosphere reserve—were identified from this extensive review by the authors of this article. For organizational purposes, the identified ecosystem services were classified according to the types of ecosystem services proposed by the Millennium Ecosystem Assessment [53]. We also used the Common International Standard for Ecosystem Services (CICES) to facilitate the description of some of the identified ecosystem services (e.g., cultural ecosystem services associated with wildlife or regulation services). Table 2 shows the ecosystem services that were identified to be presented to the participants in the interview.

**Table 2:** Ecosystem services identified from an extensive literature review.

Type	Services
Provisioning	Food derived from traditional agriculture
	Food derived from organic agriculture
	Food from cattle (milk, meat)
	Forage (trees and shrubs that are useful for cattle/browse)
	Food from hunting (hunting of wild animals for human consumption)
	Mushroom hunting for human consumption
	Beekeeping
	Wild fruits (for human and animal consumption)
	Medicinal plants (leaves, bark, roots)
	Genetic resources (e.g., wild species used in breeding programmes)
	Seeds
	Plants for fibres/handcrafts
	Industrial use of animals and plants
	Drinking water
	Water for agriculture
	Water for industrial use
	Wood fuel
	Coal
	Wood for building
	Organic compost
	Soil litter extraction
Regulating	Genetic pool of the plant communities in central Chile with global relevance
	Fresh air and climate change control
	Soil fertility for agricultural crops and pasture
	Water regulation and retention
	Erosion control
	Pest and disease control
	Pollination
Cultural	Educational value: possibilities of developing educational programmes about local wildlife
	Conservation activities carried out for different actors motivated by iconic threatened animal and plant species (conservation value)
	Rural tourism
	Resort tourism
	Cultural tourism
	Nature tourism
	Sport hunting
	Possibilities to develop research (e.g., genetic patterns in plants, effects of invasive species on the dynamics of Chilean palm relicts)
	Local ecological knowledge
	Identity and sense of place
	Spiritual and religious values
	Symbolic animals
	Symbolic plants

Given the scarce information on different local stakeholder preferences for ecosystem services in the study area, our study had a consultative participatory characteristic. Consultative social studies are suggested when there is a lack of information on the topic of interest [25]. We used a semi-structured interview approach to explore the preferences of the local actors for the ecosystem services listed in Table 2. Semi-structured approaches can be useful when starting research on a social topic on which there is not much information (e.g., [7]). In addition, we chose a semi-structured interview approach, as this method has been recognized as useful when the goal is to characterize social actors according to their perceptions about ecosystem services and use this information to provide initial images of diverging interests among stakeholders for ecosystem services when such images do not exist, such as in the case of our study [24,39]. Furthermore, the use of a semi-structured interview allowed us to incorporate social perceptions of nature and make them compatible with the definitions of ecosystem services, which is a category that is understandable for professionals of both the natural and the social sciences. Another important advantage of a semi-structured approach is that it assesses the social perception of ecosystem services using the same subjective rating scale for services with market value (fundamentally those of supply or some cultural type, such as tourism) or without market value (most regulating services and cultural services of rural type as the cultural identity). Thus, this approach can incorporate a wide spectrum of links between society and nature at the same level of analysis [39]. The proposed method allows us to relate different ecosystem services with different social actors and to start identifying diverging interests related to ecosystem services; as a result, this approach greatly facilitates planning processes and paves the way for the use of methodologies, such as group valuation (e.g., [54]), deliberative valuation (e.g., [5]) or in-depth interviews (e.g., [26]), that pose more interactive participatory techniques and benefit from the results of interviews like the one presented here. Thus, the use of a semi-structured interview, in addition to in-depth interviews and other methods of deliberative participation, contributes a direct component to the evaluations, making this approach a suitable method that can be applied to a broad spectrum of people who do not always feel comfortable with techniques that involve group participation or long conversations [39].

The interview structure was as follows (S1 Questionnaire): First, the interview included an introductory section that explained the following aim: to learn about their opinions on nature of the place to inform the decision-making process related to ecosystem management. To accentuate neutrality, we took careful consideration to avoid introducing a “pro-conservation” subtext in the questionnaire [55]. Then, we asked the respondents to choose and rank the 5 most important ecosystem services from the list (Table 2). When presenting this question, we did not explicitly use the concept of ecosystem services; rather, we asked the respondents to choose the “five things that they use, like or value most”. We explained that these things could be material (e.g., food obtained from agriculture) or non-material (e.g., identity and sense of place). To further explore the reasoning behind the stated preferences for ecosystem services, for each of the 5 most important selected services, we asked the respondents to explain why each service was important to them through the use of an open question. Next, we asked about the perceived vulnerability of the chosen services to negative changes in the future and explained that vulnerability refers to the risk of the loss of quality or the disappearance of an ecosystem service. Respondents gave each selected service a vulnerability score using a five-point Likert scale [56] (i.e., from 1, not at all vulnerable, to 5, extremely vulnerable).

The last section of the interview asked about the sociodemographic characteristics of our participants, such as their occupation, educational level, gender, age, rural-urban personal feeling and duration of residence in the place. We also asked respondents about their environmental attitudes and how they learned about nature (e.g., type of knowledge of nature) and about their environmental attitudes (e.g., whether the respondents knew about the types of protected areas, whether they visited protected areas or natural spaces, whether they practised recycling and whether they were members of environmental associations). Interviews were conducted by 3 anthropologists (one of which is a co-author of this article) who had experience in socio-environmental contexts and who presented themselves to respondents as collaborators of a scientific project conducted by the Faculty of Forest Sciences and Conservation of Nature of the University of Chile. During the application of the questionnaire, we sought to generate a bond of trust with the people who were interviewed; thus, the fieldwork experience of the anthropologists contributed to the appropriate implementation of the interview. Participants were identified through key contacts and through snow-ball sampling, and they were contacted by the research team by e-mail, telephone and in person. A total of 70 in-person semi-structured interviews were conducted in January 2016 with the key local actors described in Table 1, and the key actors were distributed among the biosphere reserve territory. The sampling strategy allowed us to collect a broad variety of perspectives regarding ecosystem services. Similar sampling strategies have been used in other studies of ecosystem services (e.g., [57]). In some cases, interviewers had to provide careful explanations about the meaning of the listed ecosystem services to the local actors. When respondents had questions on the meaning, the surveyors used the same definitions for the ecosystem services provided on the list. Additionally, locally appropriate examples were offered if needed. Such definitions were raised during the design of the interview. We also recorded the interviews in order to collect all information given in the discourses and to facilitate data computing.

#### Data analysis

To analyse the relative importance that different stakeholders attribute to different ecosystem services, we obtained a weighted sum by transforming the ordinal data from the ranking into quantitative data for each service. This weighted sum was obtained by multiplying the frequencies by which a service was placed in each position, e.g., the service ranked as most important was weighted by 5, the second by 4, the third by 3, the fourth by 2 and the fifth by 1, and then all the values were summed. To assess the perceived vulnerability of each service, we calculated the mean for each using all the vulnerability values attributed to each service. To explore the association of the stakeholders’ appreciation of the importance of the various ecosystem services with the sociodemographic and cultural characteristics of the respondents and to identify contrasting perceptions among stakeholders, we used the vulnerability and importance values of the 41 ecosystem services and then calculated an importance-vulnerability index for each service to select those that were most valued by the respondents. The index consists of multiplying the weighted sum of importance by the mean vulnerability for each ecosystem service. Ecosystem services that presented an importance-vulnerability index value that was higher than the mean were selected for statistical analysis. Importance values were normalized by calculating the z-scores and analysed by principal component analysis (PCA) [57,58], and the components were chosen using the Kaiser criterion (eigenvalue higher than 1) [57,59]. To identify the main sociodemographic variables and cultural characteristics of respondents that guide perceptions of ecosystem services, we chose the variables that obtained the largest square cosine in the selected components [57].

The selected variables were analysed using redundancy analysis (RDA) [6,7]. RDA allows response variables (in this case, the importance values of the ecosystem services calculated from the ranking by the respondents) to be related with a set of predictors of interest [60]. A Monte-Carlo permutation test (1,000 permutations) was performed to determine the significance of the independent variables in explaining the importance scores of the selected ecosystem services [6,7]. The most important variables in explaining respondent perceptions of the importance of ecosystem services were identified based on their factor scores [7]. Hierarchical cluster analyses were used to better visualize the preferences for ecosystem services and their relations to stakeholders [6].

Answers to the open question that asked the respondents to provide justification for the value of importance they placed on the 5 selected ecosystem services were transcribed verbatim into an Excel database, and these responses guided the inductive identification of the different arguments that the participants used to explain why the selected ecosystem services were important to them. Arguments were then classified into values using an adapted value typology derived by Rolston and Coufal ([61]; Table 3). This value typology has been linked to ecosystem services frameworks in different studies (e.g., [62–64]) which highlight its utility for land planning and management purposes.

**Table 3:** Value typology used to classify the justifications of importance of the ecosystem services given by respondents. Modified from Rolston and Coufal [61].

Value	Description Arguments of importance of the ecosystem service given by respondents are related to:
***Aesthetics/Scenic***	Scenic beauty, smells or sounds
***Economic***	The possibility of exchanging the service for money
***Education/Scientific research***	The possibility of learning about nature through observation and study
***Biological***	The variety of animals, plants, and other living organisms that can be valued because they can directly be used by humans or because they have ecological importance
***Spiritual***	Mystic, religious or spiritual importance
***Cultural Identity***	Traditions that depend on the ecosystem service. These traditions contribute to local livelihood and sense of place
***Ecological/Environmental***	Ecological importance and maintenance of ecosystem functionality and resilience and conservation of biodiversity
***Recreation***	Possibilities of outdoor recreational activities
***Scarcity***	Threats to the ecosystem service flow and decay processes of the service
***Personal benefit***	An auto-satisfaction process and personal development. Not related to the possibility of exchanging the service for money

The codifications were agreed between three of the authors of the manuscript in order to obtain a homogeneous criteria for the classification of the arguments. In the case that an argument given by the participants contained more than one orientation of value, all emerging categories were considered to encompass all the dimensions of the argument. In case of disagreement between those who codified, a category was agreed upon.

## Results

### Quantitative results

Table 4 shows overall results of importance of ecosystem services. Importance values for each service correspond to the weighted sum by transforming the ordinal data from the ranking into quantitative data. The most important service was drinking water (importance value = 124), followed by fresh air and climate change control (90), the genetic pool of the plant communities in central Chile (89), educational value (e.g., possibilities of developing educational programmes and books about local wildlife) (89), conservation activities motivated by iconic threatened animal and plant species (79) and water regulation and retention (74).

**Table 4:** Ranking of ecosystem services according to importance score, mean vulnerability value and importance-vulnerability index. The order of ecosystem services in the first column follows the importance-vulnerability index value from highest to lowest.

Ecosystem service	Importance value	Mean vulnerability	Importance-vulnerability index
Drinking water	124.0	4.0	498.7
Fresh air and climate change control	90.0	3.9	352.1
Conservation activities motivated by iconic threatened animal and plants species	79.0	4.4	349.3
Genetic pool of the plant communities in central Chile with global relevance	89.0	3.7	332.1
Water regulation and retention	74.0	4.3	321.4
Educational value: possibilities of developing educational programmes about local wildlife	89.0	2.7	240.0
Water for agriculture	49.0	3.6	176.4
Food derived from traditional agriculture	36.0	4.1	145.8
Medicinal plants (leaves, bark, roots)	39.0	3.5	136.5
Symbolic plants	32.0	3.6	116.5
Beekeeping	33.0	3.5	113.9
**Mean**	**108.2**		
Identity and sense of place	38.0	2.7	100.7
Food derived from organic agriculture	36.0	2.8	100.2
Local ecological knowledge	24.0	3.8	90.4
Erosion control	31.0	2.7	84.2
Food from cattle (milk, meat)	24.0	3.4	80.4
Forage (trees and shrubs that are useful for cattle/browse)	18.0	3.4	61.2
Nature tourism	34.0	1.8	60.7
Possibilities to develop research	21.0	2.2	46.2
Spiritual and religious value	14.0	2.0	28.0
Rural tourism	19.0	1.3	25.3
Wild fruits (for human and animal consumption)	14.0	1.5	21.0
Soil fertility for agricultural crops and pasture	8.0	1.7	13.7
Seeds	9.0	1.4	12.6
Symbolic animals	7.0	1.8	12.6
Organic compost	8.0	1.5	12.0
Pest and disease control	9.0	1.3	11.7
Pollination	5.0	1.6	8.0
Genetic resources (e.g., wild species used in breeding programmes)	3.0	1.8	5.4
Water for industrial use	5.0	1.0	5.0
Cultural tourism	5.0	0.8	4.0
Wood fuel	4.0	0.6	2.4
Industrial use of animals and plants	5.0	0.4	2.0
Plants for fibres/handcrafts	0.0	0.0	0.0
Food from hunting	0.0	0.0	0.0
Sport hunting	0.0	0.0	0.0
Coal	0.0	0.0	0.0
Wood for building	0.0	0.0	0.0
Resort tourism	0.0	0.0	0.0
Mushroom hunting for human consumption	0.0	0.0	0.0
Soil litter extraction	0.0	0.0	0.0

The services with the highest perceived vulnerability scores were the conservation value of threatened animal and plant species ($\bar{x}$ = 4,421), water regulation and retention ($\bar{x}$ = 4,343), food from agriculture ($\bar{x}$ = 4,050), and drinking water ($\bar{x}$ = 4,022) (Table 4).

**Fig 2:**
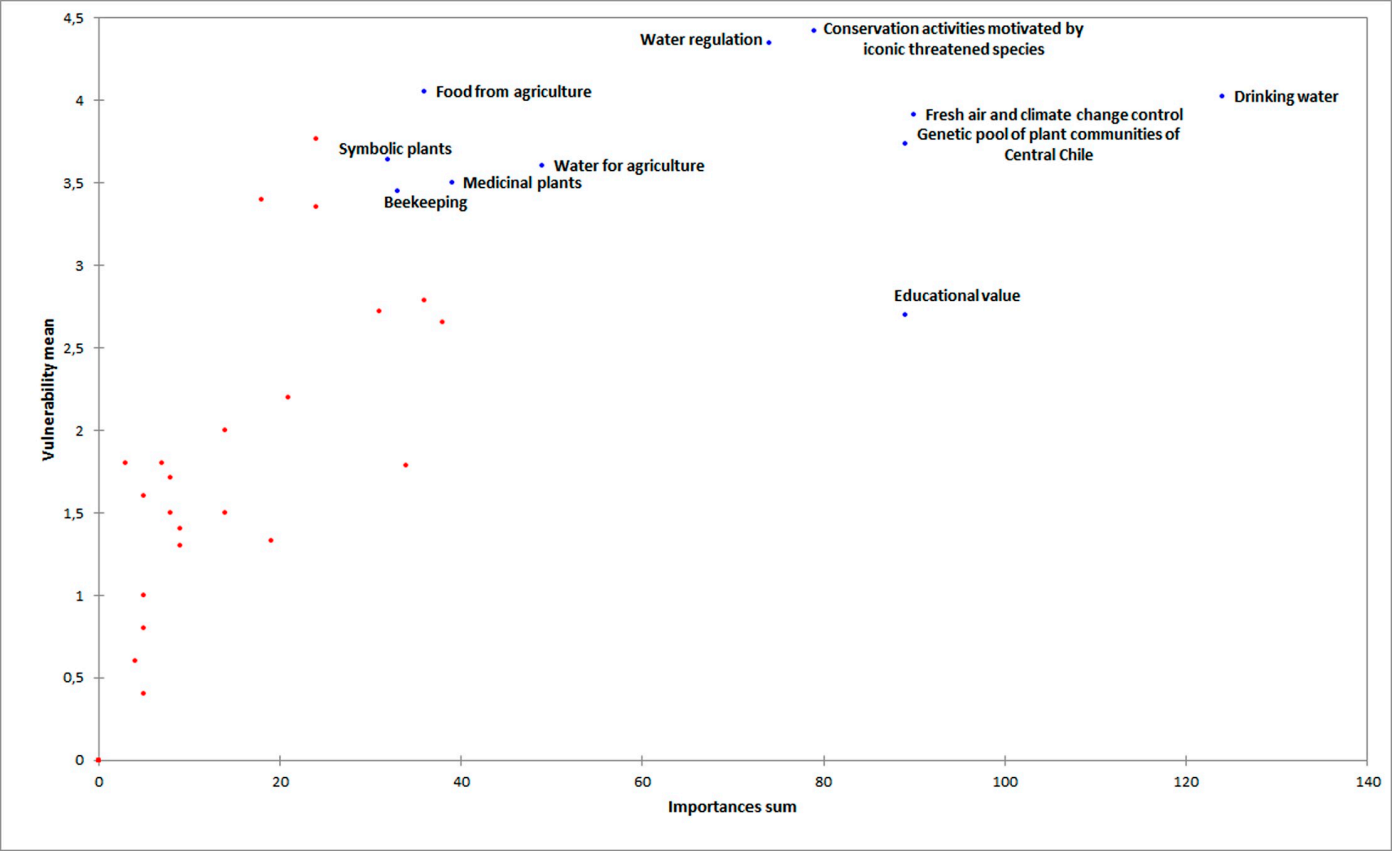
Graphical representation of the 11 services selected for analysis and their importance and vulnerability scores.

The ranking of ecosystem services shows that 11 services have higher-than-mean importance-vulnerable index scores ($\bar{x}$ = 108.2; Table 4). These 11 services were used to analyse the association between the appreciation of ecosystem services and the sociodemographic and cultural characteristics of the respondents, and this information was used to identify contrasting perceptions of stakeholders regarding such services. Fig 2 shows a graphical representation of the 11 services selected for analysis and their importance and vulnerability scores. These services were then analysed using PCA, and the services with the largest square cosine in those components with an eigenvalue greater than 1 were finally selected to explore their association with the sociodemographic and cultural characteristics of the respondents, and to identify contrasting perceptions of stakeholders regarding the ecosystem services (S1 Table).

The Supplementary Information (S1 Table) presents the factor scores resulting from RDA. All variables used for the analysis can be seen in the Supplementary Information (S2 Table).

**Fig 3:**
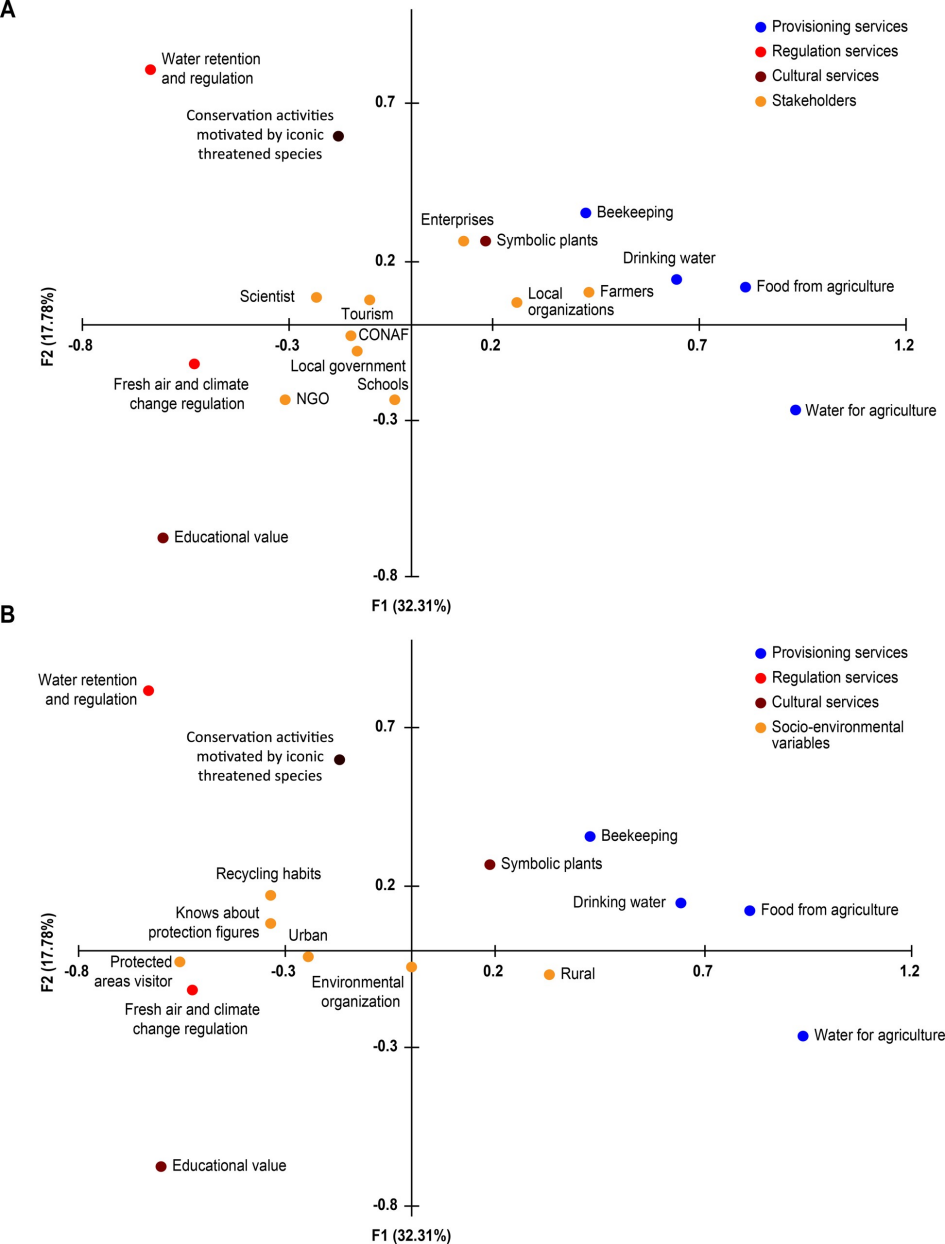
(A, B). RDA Biplot. To better visualize the results, the graphic was divided into two separate figures. Both were created with the results of the same analysis using all variables.

Fig 3A and 3B presents the biplots obtained from RDA. To better visualize the results, we divided the graphic into two separate figures. RDA indicates a significant association between the characteristics of the stakeholders (e.g., occupation, feeling of being rural-urban, whether they visit protected areas, whether they have knowledge of protection figures, membership in environmental organizations and recycling habits) and the relative importance of ecosystem services as perceived by locals (p = 0.044, 1,000 permutations). The first 5 axes explained 83% of the total variance (S1 Table), and based on the explained variance and eigenvalues, we focused on the first 2 axes because they showed the most important trends in terms of explaining the differences in stakeholders’ perceptions of important ecosystem services.

**Fig 4:**
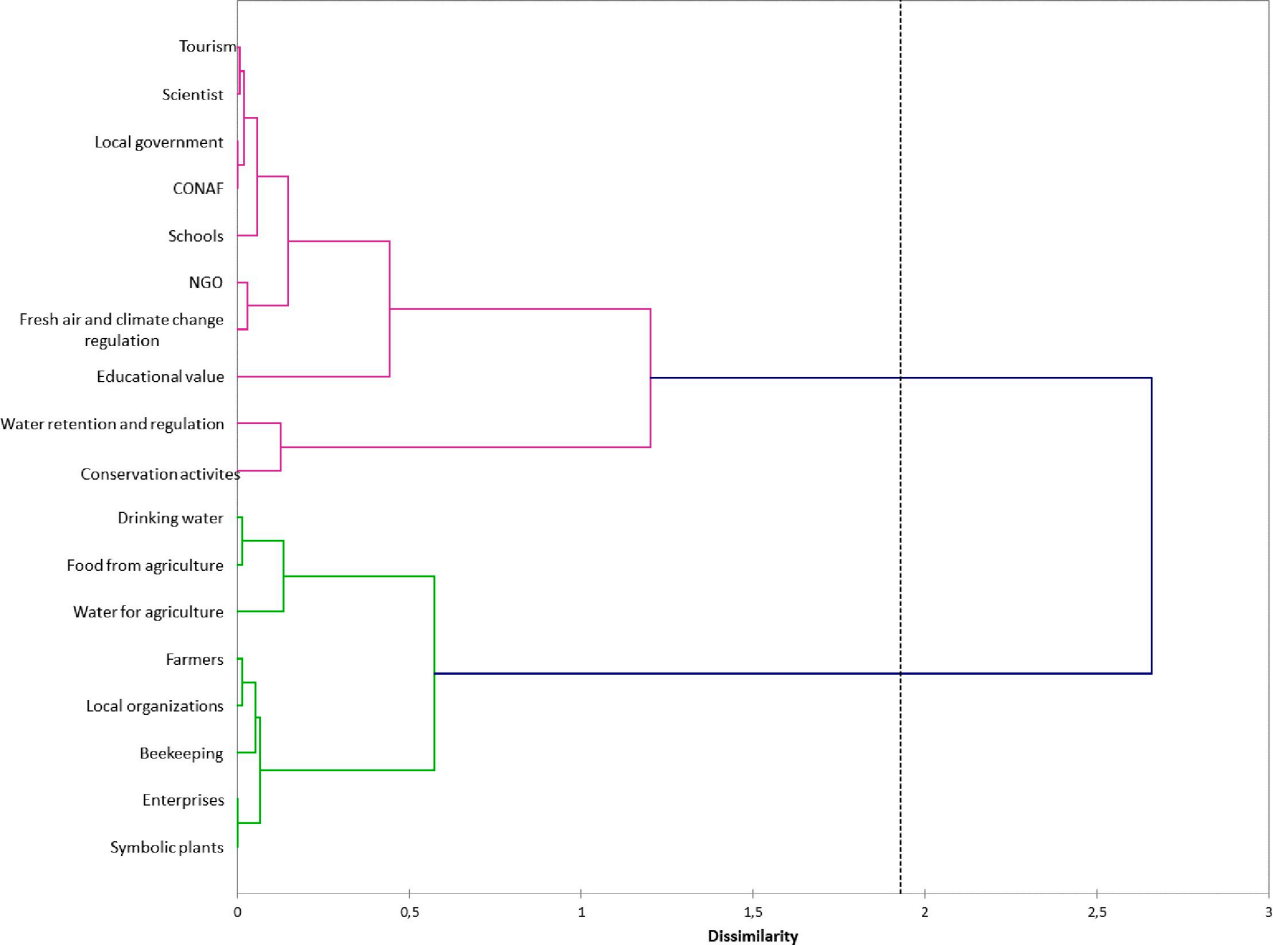
Dendrogram from the hierarchical cluster analysis.

Fig 3A shows the associations between the ecosystem services and the variable occupation. The F1 axis (horizontal) explained 32.31% of the total variance, showing contrasting perceptions of importance between provisioning services (e.g., food derived from traditional agriculture, water for agriculture, drinking water and beekeeping) and symbolic plants (a cultural service) on the positive side of the axis, and this result was juxtaposed against water regulation and conservation activities (on the negative side of axis 1 and the positive side of axis 2) and educational value on the negative sides of axes 1 and 2. We found that occupation affected the different perceptions regarding the importance of ecosystem services. Small farmers, members of local organizations, and managers/owners of enterprises gave higher values of importance to provisioning services and symbolic plants, while NGO members, employees of the National Forest Corporation (CONAF), and employees of the local government and schools gave higher values of importance to fresh air and climate change control and educational value; furthermore, scientists and tourism workers preferred water regulation and retention and the possibilities of developing conservation activities motivated by threatened wildlife. The F2 axis (which explained 17.78% of the variance) shows contrasting perceptions between the perceived importance of water retention and regulation and the possibilities of developing conservation activities juxtaposed against the educational value and fresh air and climate change control. The characteristics of stakeholders and respondents were not strongly associated with their preferences for ecosystem services on this axis, indicating that there must be some variables that explain this dichotomy that were not included in our study. The hierarchical cluster analysis (Fig 4) provided a better visualization of this dichotomy. This analysis distinguished between two main groups of stakeholders based on their contrasting preferences for the ecosystem services described above.

Fig 3B shows the associations between the ecosystem services and the other sociodemographic and cultural characteristics of respondents that were independent of occupation (e.g., rural-urban and whether the respondents visit protected areas, have recycling habits and have knowledge of protection figures). Respondents who self-identified as rural more often preferred provisioning services and beekeeping, but respondents who self-identified as being urban as well as respondents who were interested in environmental protection topics or had some information on conservation aspects (defined as those who recycle, know about protected areas, visit protected areas and are members of environmental organizations) placed more importance on water retention and regulation, fresh air and climate change control, conservation activities related to threatened species and educational value.

### Qualitative results

Table 5 shows the results of the open-ended question that was used to evaluate why the five selected ecosystem services from the list (Table 2) were important to the respondents. Arguments are classified into value dimensions of the typology derived by Rolston and Coufal ([61]; Table 3). Percentages in bold correspond to the value dimensions that were most frequently mentioned. In the last column, we provide examples of arguments supplied by respondents in relation to the most frequent value dimensions.

**Table 5:** Arguments supplied by respondents to explain why the ecosystem services were important to them, classified according to the value dimensions derived by Rolston and Coufal [61].

Ecosystem services	Aesthetics	Economic	Education/research	Biological	Spiritual	Cultural identity	Environmental / Ecological	Recreational	Scarcity	Personal benefit	Example of reasons given for justifying the importance of ecosystem services
**Food derived from traditional agriculture**	0%	**41%**	0%	0%	0%	**18%**	0%	0%	0%	**41%**	Economic and personal benefit: "*For sale and auto-consumption*", Cultural identity: “*Way of life that should be preserved*”.
**Symbolic plants**	0%	5%	11%	**21%**	5%	**26%**	**21%**	0%	0%	11%	Cultural identity: "*Vegetation has a patrimonial value*", "*plants maintain traditions*”, “*school children should know and value plants as a form of identity*". Biological: “*Endemic plants contribute to hydrological cycle*”.
**Drinking water**	0%	2%	10%	7%	0%	7%	**15%**	0%	**34%**	**24%**	Scarcity and personal benefit: “*We have had the well for 14 years and have spent entire seasons without water*”, Scarcity: “*the drought has lasted 5 years*”, “*It is vital especially now because of the drought*”. Environmental: “*The absence of water will cause disequilibrium*”, “*we should avoid drying plants and trees*”.
**Water for agriculture**	0%	**37%**	5%	0%	0%	0%	5%	0%	**53%**	0%	Scarcity: "*little precipitation*", "*scarce resource*". Economic: "*last year we planted half less due to drought*".
**Water regulation**	0%	**11%**	0%	**26%**	0%	7%	**48%**	0%	7%	0%	Environmental: "*plant and animal communities depend on this service*", "*flora and fauna need water*". Environmental: "*Mediterranean forests maintain water*". Economical: “*Agricultural use*”.
**Conservation value of threatened animal and plant species**	3%	**10%**	7%	**37%**	0%	7%	**30%**	0%	7%	0%	Biological: "*Because there are unique species that have been recognized as endangered species*". Environmental: "*Conservation for balance*, *nature is balance*, *anything against nature triggers an imbalance*". Biological: "*to maintain a genetic pool*". Economic: “*well-preserved components such as animals and plants give an aggregated value to the area for the development of different activities (e*.*g*., *tourism)*”.
**Fresh air and climate change control**	3%	0%	10%	6%	0%	0%	**48%**	0%	0%	**32**%	Environmental: "*Desertification is slowing down native species conservation*", "*the earth requires an oxygen lung* ". Personal benefit: "*delicious forest air*", "*breathe fresh air*", "*clean lungs*".
**Beekeeping**	0%	**25%**	0%	13%	0%	6%	**25%**	0%	6%	**25%**	Environmental: "*It is linked to the ecosystem*, *without this service the environment could not be developed*", "*pollination benefit*". Economic: “*economic support*”, “*Profitable sources of income*”. Personal benefit: “*I like honey*, *I like the local honey production*”.
**Educational value**	0%	0%	0%	**16%**	0%	**20%**	**64%**	0%	0%	0%	Environmental: "*To know the ecosystem where we live*", Biological: "*To learn about birds and the environment*", “*plants and animal recognition*”, "*Education for conservation*". Cultural identity: "*Education about the rural environment*", "*It is linked with identity and relations between people and their territory*".

## Discussion

The necessity of exploring the social values that different stakeholders attribute to ecosystem services has been widely recognized [6,65]. In our study, different actors revealed contrasting preferences for services. In this context, our research has allowed us to link different types of actors with different ecosystem services, and this approach can facilitate subsequent planning and decision-making processes that pave the way for the use of methods that involve more interactive and deliberative participation [5]. In addition, the justifications of the importance of ecosystem services provided by the participants shed light on the perceptions that guide the values placed on different ecosystem services. In this regard, our work, despite its consultative nature, provides elements that allow us to the understanding of the diversity of orientations of value associated with benefits of nature in Chile.

We found that the most-preferred ecosystem services were drinking water, fresh air and climate change control, the genetic pool of the plant communities in central Chile, educational value, possibilities of developing conservation activities motivated by iconic threatened animal and plant species, and water regulation. Preferences differed among stakeholders and were influenced by different characteristics, such as occupation, knowledge about protection figures, rural-urban feeling, whether respondents belong to an environmental organization, whether they are visitors of protected areas and whether they have recycling habits. Provisioning services were preferred more by small farmers, local organizations and enterprise managers/owners, and these groups also preferred cultural ecosystem services related to plants. In addition, stakeholders who identified themselves as rural actors tended to prefer provisioning services. More urban and formally educated stakeholders (e.g., scientists, NGO members, local government employees, tourism managers) preferred regulating and cultural services (e.g., possibilities for developing conservation activities focused on threatened animals and plants). Some studies have shown that these contrasting perceptions of ecosystem services are present in agro-ecosystems [7,66] as well as in biosphere reserves [57]. For most interviewees, the most important ecosystem service was the provision of drinking water, which is understandable due to the semi-arid climate and the severe drought that has affected the region since 2010 [67]. However, local farmer organizations and stakeholders that self-identify as rural seemed to be more concerned about the provision of this service than were other respondents. This difference was likely because of their direct dependence on this service. In their narration of the justification of importance, the drought experienced by respondents was one of the main reasons why this service was highly valued. For example, some respondents revealed that they have had entire seasons without water. Thus, the higher values given to this service by local stakeholders could be explained by the fact that they recognize the importance of this service in the maintenance of their livelihoods [68].

Food production from agriculture was also more important for this group of local actors, who claimed they received economic and personal benefits from the service. It is remarkable that a group of people also assigned importance to this service due to the recognition of the fact that their identity has been forged by their relations with agricultural products. In recent decades, the practice of small-scale agriculture has decreased in the biosphere reserve territory because of drought, urbanization, migration and lack of governmental politics and this decline undermines the reproduction of their culture and way of life. This deterioration of the cultural value associated with traditional farming activities was also recognized by the respondents. This result suggests that food from agriculture can be recognized as both a provisioning service and as a cultural service [5,68]. In this regard, future research should provide an in-depth exploration of the socio-cultural context in which local actors obtain these provisioning services. Ethnographic research could contribute to better understanding whether food production from agriculture is mostly based on an utilitarian perspective and on how socio-cultural aspects such as local identity influence the way locals develop agricultural activity.

Scientifically or formally educated people (in our study these respondents were mainly represented by scientists, CONAF employees, educators, and employees of the local government) more often recognized the key importance of regulating services, such as water regulation, as well as the scientific and educational value of ecosystems. Usually, people living in cities have a higher average level of formal education and have left behind the rural life. This urban lifestyle can cause them to become disconnected from the ecological basis of material production, and the food and water supply can be taken for granted and are not a matter of daily concern [6].

Environmental and ecological value dimensions emerged from respondents when arguing the importance of water regulation. Arguments of importance provided by respondents did not have a clear link to human well-being; rather, there was a stronger link with ecological functioning. The fact that respondents also value water regulation because it is important for flora and fauna may reveal an existence value dimension of species (i.e., the satisfaction of knowing that a species exists: Krutilla [69]; see also Cerda et al. [70]), and this value could motivate respondents to attribute importance to this service.

Additionally, the fact that respondents with a higher level of formal education valued regulating services more than did actors whose knowledge came from direct experiences with nature makes our results different from those reported in other studies. For example, in Spain, Martin-López et al. [6] found that the local ecological knowledge was bundled with regulating services related to water and soil, and the authors argued that such a result may be because the most traditional land management practices focus on managing these services to tackle soil erosion, aridity, drought, and flooding. Over time, the biosphere reserve in which our study was focused has been characterized by a high diversity of land uses that have continuous change dynamics, and this characteristic has resulted in an urban-rural population that is increasingly mobile [21]. Such mobility may affect the preservation of local ecological knowledge that could be used to visualize regulating ecosystem services. In this regard, complementary research that uses additional strong participatory approaches, such as deliberative participatory techniques [5], should be used to more deeply analyze how the land management practices that are implemented by locals consider regulating services.

The educational value of ecosystems was valued more by respondents with scientific or formal education, and similar results have been found in other studies (e.g., [6]). Arguments that justify its importance are related to ecological and cultural value dimensions, although cultural values were mentioned less by respondents. In the case of ecological arguments, respondents saw education as being necessary for achieving conservation goals. In addition, from the perspective of this group of respondents, birds emerged as a taxonomic group that offered education possibilities. Other studies in Chile (e.g., [71]) and at international level (e.g., [72]) also found that birds provided motivation to urban and formally educated citizens to apply for public funds for conservation projects.

Interestingly, members of the group of formally educated respondents saw the link between local people and their surrounding ecosystems in terms of educative value. In their arguments, people may be capturing the importance that they attribute to retaining traditional local practices of land use. In this regard, the way in which environmental education programmes are transmitted to the public should be carefully designed in the study area. Such a design should consider human needs, particularly those of subsistence. In addition, local ecological knowledge should be considered, given that biodiversity supports a broad range of cultural practices and adaptations that may be decisive for achieving conservation goals [73]. Several examples in the literature support the relevance of local ecological knowledge, which in some cases, when combined with the key involvement of external organizations, can facilitate the achievement of conservation goals (e.g., [74]). The importance given to education was based on biological and cultural values, and this information can be used to strengthen the comprehension of the cultural importance of biodiversity to different sectors of society. Additionally, these results offer great possibilities for future research that could identify which links between people and their surrounding ecosystems should be preserved and transmitted in educational programmes.

Symbolic plants were more important for farmers, local organizations and actors of local enterprises than for scientists, tourism operators, employees of local government, NGO members, employees of the Chilean National Forest Corporation (CONAF) and educators in schools and colleges. Respondents provided direct quotations such as “*vegetation has a patrimonial value*”, “*plants maintain traditions*” or “*school children should know and value them as a form of identity*”, and these statements indicate that plants provide important cultural benefits in the area. In addition, the character of a species being endemic (mainly for scientists and educators) or native also motivated the importance that people attributed to this service.

Our findings are in line with ethnographic research (e.g., [75]) that has found that many social groups have a strong relationship with plants. The cultural value attributed to plants in conjunction with endemism characteristics can be used to engage people in plant conservation.

The services perceived as more vulnerable were the possibility of continuing with conservation activities motivated by threatened wildlife, water regulation, food from agriculture, and drinking water. These results revealed a noticeable concern about threatened wildlife, which were seen as being important because of their unique character, importance for the maintenance of the natural balance, and because such species contribute to the genetic pool. These arguments allow visualizing a more biocentric view of species conservation from respondents. The fact that this service was perceived as vulnerable may indicate that communication about conservation problems has been effective and has contributed to an increased awareness by local residents regarding the conservation problems found in this area. It is also likely that the proximity of the respondents’ homes to the protected area may influence awareness with respect to the importance of protecting biodiversity.

The high vulnerability values given to food from agriculture and drinking water services could be related to the lack of water in the socio-ecological system and the degraded water quality in the study area.

In sum, scientists and employees of CONAF and the local government showed convergent interests due to their high levels of scientific and urban education, and these groups typically have more influence in policy-making than do local farmers and local organizations. Thus, the divergence of interests and the greater power of decision makers could cause the biosphere reserve model to not incorporate local demands, which could cause conflicts between locals and managers/policy makers, thereby compromising local participation in conservation initiatives and affecting the success of nature protection in the area. Unfortunately, there is no easy solution that will satisfy all stakeholders. In this regard, stronger participatory valuation techniques, such as group valuation [54], could contribute to elucidating possible solutions for sustainable management in the biosphere reserve. In this process, our study provides useful information for shaping policies based on ecosystem services. Initially, water management seems to be a key factor in resolving the trade-offs among food and water security, economic development, and regulating and cultural services [67]. Ecosystem-based approaches to water management can be integrated into agricultural development as well as biodiversity conservation [66]. However, more research is required in this area to identify a win-win solution for all stakeholders and to build resilience in the region.

Studies such as the one presented here can help identify the different interests of stakeholders as well as the winners and losers in management decisions resulting from changes in the provision of ecosystem services in the future [76]. This type of analysis of social values provides useful information for designing management plans for biosphere reserves. We have shed light on the different interests involved in ecosystem services, and our findings can contribute to visualizing the impacts of different management options on the biosphere reserve and how these options will affect the fluxes of ecosystem services for different stakeholders. In addition, our findings can be complemented with deliberative participatory methods [5,54] and aid in conflict resolution, as conflicts frequently emerge due to the different interests of stakeholders.

In many countries of the world, social valuations of ecosystems services are often ignored in political spheres and in territory management [31]. Given the market economic model that prevails worldwide, the economic valuation of ecosystem services is preferred, and it is argued that demonstrating the economic values of the benefits provided by ecosystems is the only way to incorporate such benefits into decision-making processes [11]. However, the necessity of developing new conceptual frameworks in which the management of ecosystem services assumes a more integral conception of well-being and quality of life, including social and cultural aspects, has been recognized [77]. In this framework, the social valuations of ecosystem services emerge as fundamental.

## Conclusion

The biosphere reserve model explicitly recognizes the necessity of integrating different actors into the design and implementation of effective mechanisms of biodiversity conservation at local, regional, national, and global scales. Accounting for social preferences for ecosystem services enables the multiple ways by which people benefit from nature to be visualized. In countries such as Chile where economic criteria are often given more weight than other ecological and socio-cultural criteria in territorial management, ignoring information generated from social approaches can hide the complex socio-ecological webs that are not necessarily visualized through existing legal regulations or policies. However, understanding these webs is key to advancing towards sustainability. This fact has important policy implications as it forces scientists and decision makers to recognize the legitimacy of the interests of local communities in nature, thus favouring a more transparent decision-making process. In this regard, our approach contributes to a better understanding of how the different social actors of a biosphere reserve in a biodiversity hotspot in South America perceive ecosystems from the perspective of the provision of different benefits through the following specific findings: a) divergent perceptions of ecosystem services emerged from different stakeholders; b) there was an urban-rural dichotomy in terms of preferences for ecosystem services; c) local ecological knowledge (e.g., that of small farmers) emphasized provisioning ecosystem services (e.g., beekeeping, food from agriculture, and drinking water) as well as cultural services associated with plants, while more expert knowledge (i.e., that of scientists or environmental professionals) leads to the favouring of regulating services (e.g., fresh air and climate change regulation) and cultural services (e.g., possibilities of developing conservation activities focused on threatened animals and plants). Thus, locals are guided by subsistence logic—the reproduction of their lives and their way of life—that is mainly linked to provisioning services, while scientists and environmental professionals perceive the benefits of natural systems on a more global scale by focusing on regulating services.

At the local level, we found that ecosystem services associated with water supply and agriculture (e.g., traditional activities such as beekeeping) and cultural services associated with symbolic plants seemed to be embedded in the perspective of local actors. Thus, this bundle of ecosystem services (e.g., [6]) seemed to be critical for local communities, where the provision of water was a key factor in the manifestation of such services. In central Chile, there is concern about the future water supply, which may be affected by the increasing human population and frequency of drought associated with climate change. In this regard, ecosystem-based approaches to water management can be integrated into agricultural development as well as biodiversity conservation.

In Chile, the inclusion of social perspectives in land management is still limited, although discourse on environmental sustainability is increasing. To advance towards sustainability, the needs and perspectives of all beneficiaries must be taken seriously to legitimize processes, avoid conflicts and design socially informed conservation policies. Our results may also contribute to the implementation of the planning model for protected areas based on open standards, which consider human well-being and ecosystem services. Because local planning processes are in full swing, our analysis provides timely information that can be used by local and regional stakeholders and decision makers to design stronger participative approaches; additionally, our results can be considered as base knowledge on local preferences towards ecosystem services.

Our study highlights the necessity of exploring social perceptions of ecosystem services to uncover the interests of different stakeholders in complex management scenarios, such as biosphere reserves, that must consider both natural and social issues.

## Ethics statement

Ethical approval for this study was obtained from the Research Ethics Committee in Social Sciences and Humanities of the Faculty of Philosophy and Humanities of the University of Chile. Anonymity and confidentiality were explicitly granted to respondents. Participants agreed to respond to the questionnaire once they had given their informed consent, which was reviewed and approved by the mentioned Ethics Committee.

This study is part of the multidisciplinary research project “Exploring human-wildlife relationships in Chile: a multi-stakeholder perspective of conservation management”, which is funded by the Chilean Science and Technology Commission for the years 2015–2018. This multidisciplinary project focuses on the socio-cultural valuation of the different animal and plant species present in the territory covered by the biosphere reserve as well as on the social valuation of the ecosystem services provided by the reserve. In this article, we focus on the social valuation of ecosystem services.

## Supporting information

S1 Questionnaire (DOCX)

S1 Table Resulting factor scores from RDA. Eigenvalues and variance explained by the analysis. Biplots were created using these data.
(DOCX)

S2 Table Results from PCA. Bold Variables did not Present Any of the Largest Square Cosine Values and were Excluded from RDA.
(DOCX)
