# Supplementary material for: Social preferences for ecosystem services in a biodiversity hotspot in South America
Source: PLoS One. 2019 Apr 22;14(4):e0215715. doi: 10.1371/journal.pone.0215715 (PMC6476511; doi:10.1371/journal.pone.0215715)
Supplement: S1 Questionnaire — (DOCX) [file pone.0215715.s001.docx]

**ASSESSING SOCIAL PREFERENCES TOWARDS WILDLIFE AND ECOSYSTEM SERVICES IN A BIOSPHERE RESERVE (LA CAMPANA – PEÑUELAS)**

**Semi-structured interview for local key stakeholders**

**(English version)**

**Fondecyt Grant N° 1151063**

| **This is a shortened version of the original interview, showing only those questions related to sociocultural valuation of ecosystem services, whose results are presented on the manuscript. The original survey included other topics about animal and plant preferences that are not included here.** |
| --- |

**SENTECES AND WORDS IN BOLD/ITALICS MUST NOT BE READ TO THE RESPONDENTS. THEY ARE INSTRUCTIONS FOR THE INTERVIEWER**

**1. INTRODUCTION**

At the Faculty of Forestry and Nature Conservation in Universidad de Chile, we are interested in knowing the linkages between nature and people, especially those living in “La Campana-Peñuelas Biosphere reserve”, like yourself (***after finishing the sentence show a map of the biosphere reserve and the interviewed's location at the moment of the interview***). This time, our main task is to identify and assess the contribution of different nature’s traits to your daily life. This interview would be very helpful to complete our task. ¿would you be interested in answering this interview? It’s completely confidential. With the information we hope to contribute to decision making processes of the biosphere reserve. Please keep in mind that there aren’t wrong or correct answers, we are interested in your opinion.

| ***Interviewer*** |  |
| --- | --- |
| ***Questionnaire nº*** |  |
| ***Location*** |  |
| ***Date*** |  |

**2. ECOSYSTEM SERVICES**

We will begin talking about the features you like, use and value in the natural ecosystems of La Campana Peñuelas Biosphere reserve:

2.1 From the following list, select the 5 things that you most use, like, or value from La Campana Peñuelas Biosphere Reserve. They can be material and immaterial things (material things are for example food or coal, immaterial are for example social cohesion). You can value these things from a purely personal perspective or thinking in collective terms. ***Provide a marker to the interviewee to mark them.***

Is there anything that is not in the list and that you use, like or value of La Campana Peñuelas Biosphere Reserve?

____________________________________________________________________________________________________________________________________________________

2.2 After selecting them, order them according to how much you benefit from each thing, where: 1: greater benefit; 5 minimum benefit.***Complete in the current table and ask about the perceived vulnerability***

| **IDENTIFIED ECOSYSTEM SERVICE**  **(Ranking of importance)** | **DEGREE OF VULNERABILITY TO NEGATIVE CHANGES IN THE FUTURE**  **(in a scale of 1, ..., 5, where 1: not at all vulnerable and 5: extremely vulnerable)**  **(*Show card*)** |
| --- | --- |
| 1. |  |
| 2. |  |
| 3. |  |
| 4. |  |
| 5. |  |

Why are the selected things important to you?

________________________________________________________________________________________________________________________________________________________________________________________________________________________________________________________________________________________________________

**3. PARTICIPANT’S INFORMATION**

***Residency:***

| 1. Location where you usually live |  |
| --- | --- |
| 2. How long have you been living in location (here / there) |  |
| 3. Do you feel? | Rural**_____**; Urban**_____** |

***Environmental behavior:***

By personal choice, are you an active member of any citizen organization or Foundation for public, agrarian, environmental or other purposes?

| 1. Yes |  |
| --- | --- |
| 2. No |  |

¿Which?

Have you visited protected areas in recent years?

| 1. Yes |  |
| --- | --- |
| 2. No |  |

How have you learned what you know about the nature of this place? ***Mention each option and ask yes or no***

| By training (talks, workshops) |  |
| --- | --- |
| By reading articles or magazines |  |
| By watching TV |  |
| By direct experience with nature |  |
| By traditions, handed down from generation to generation |  |
| College Degree or related (University Education) |  |
| Other: |  |

Do you separate waste, reuse or recycle?

| 1. Yes |  |
| --- | --- |
| 2. No |  |

***Sociodemographic questions:***

| Age (years) |  |
| --- | --- |
| Gender | F:_____; M:_____ |
| Profession and activity / occupation |  |

*Level of education*:

| No education |  |
| --- | --- |
| Primary School |  |
| Secondary School |  |
| College/University |  |
| Post Graduate Education |  |

Are there any other person(s) do you consider relevant to contact to answer this interview? ***(ask for contact information)***

Do you want to ask a question or comment? How could this instrument be improved?

**THANK YOU VERY MUCH FOR YOUR HELP**

**EVALUACIÓN DE PREFERENCIAS SOCIALES POR VIDA SILVESTRE Y SERVICIOS ECOSISTÉMICOS**

**EN LA RESERVA DE BIÓSFERA LA CAMPANA PEÑUELAS**

**Entrevista semi-estructurada para Actores Locales Clave**

**(Versión en Español)**

**Proyecto Fondecyt N° 1151063**

| **Este es un extracto de la entrevista original aplicada. Aquí se presentan las preguntas realizadas sobre valoración sociocultural de servicios ecosistémicos cuyos análisis se presentan en el manuscrito. El cuestionario contuvo también otras partes que indagaron sobre tópicos diferentes como la valoración de animales y plantas del lugar que no fueron abordadas aquí.** |
| --- |

**PALABRAS Y FRASES EN NEGRITA/CURSIVA NO DEBEN SER LEÍDAS AL ENTREVISTADO(A). CONSTITUYEN INSTRUCCIONES PARA EL ENTREVISTADOR**

**1. INTRODUCCIÓN**

En la Facultad de Ciencias Forestales y de la Conservación de la Naturaleza de la Universidad de Chile nos interesa conocer los vínculos de las personas que viven en la Reserva de Biósfera La Campana Peñuelas con la naturaleza ***(mostrar mapa de la Reserva dejando en claro los límites y el lugar donde se encuentra el entrevistado(a) al momento de la entrevista)***. En esta ocasión estamos identificando y analizando las contribuciones que diferentes atributos de la naturaleza aquí tienen para Usted. Nos será de gran ayuda conocer su opinión en esta entrevista. ¿Está interesado en responderla?, la entrevista es totalmente anónima. Con la información pretendemos contribuir con los procesos de toma de decisión de la reserva de biósfera. No hay respuestas correctas o incorrectas, nos interesa su opinión.

| ***Encuestador(a)*** |  |
| --- | --- |
| ***N° encuesta*** |  |
| ***Localización*** |  |
| ***Fecha*** |  |

**2. SERVICIOS ECOSISTÉMICOS**

Primero hablaremos de lo que usted usa, le gusta o valora de la naturaleza de la Reserva de Biósfera La Campana Peñuelas:

2.1 Del siguiente listado seleccione las 5 cosas que Usted más usa, le gustan, o valora de la Reserva de Biósfera La Campana Peñuelas. Pueden ser cosas materiales e inmateriales (cosas materiales son por ejemplo alimentos o carbón, inmateriales son por ejemplo la cohesión social). Usted puede valorar esas cosas desde una perspectiva puramente personal o pensando en términos colectivos. ***Proporcionar plumón al entrevistado para que las marque.***

¿Hay algo que no esté en el listado y que Usted use, le guste o valore de la Reserva de Biósfera La Campana Peñuelas?

____________________________________________________________________________________________________________________________________________________

2.2. Luego de seleccionarlas, ordénelas según cuánto se beneficie de cada cosa, donde:

1: mayor beneficio; 5 mínimo beneficio. ***Completar en la tabla actual y preguntar por la vulnerabilidad percibida.***

| **SERVICIO ECOSISTÉMICO IDENTIFICADO**  **(Ranking de importancia)** | **GRADO DE VULNERABILIDAD A CAMBIOS NEGATIVOS EN EL FUTURO**  **(en escala de 1, …,5; donde 1: para nada vulnerable y 5: extremadamente vulnerable)**  ***(Mostrar tarjeta)*** |
| --- | --- |
| 1. |  |
| 2. |  |
| 3. |  |
| 4. |  |
| 5. |  |

¿Por qué son las cosas seleccionadas importantes para Usted?

________________________________________________________________________________________________________________________________________________________________________________________________________________________________________________________________________________________________________________________

**3. DATOS DEL PARTICIPANTE**

***Residencia:***

| 1. Lugar donde vive habitualmente |  |
| --- | --- |
| 2. Tiempo viviendo en el lugar (aquí/allí) |  |
| 3. Usted se siente: | Rural**_____**; Urbano**_____** |

***Comportamiento ambiental:***

Por opción personal, ¿es Usted es miembro activo de alguna Organización ciudadana o Fundación con fines públicos, agraria, ambiental u otra?

| 1. Sí |  |
| --- | --- |
| 2. No |  |

¿Cuál?

¿Ha visitado áreas protegidas en los últimos años?

| 1. Sí |  |
| --- | --- |
| 2. No |  |

¿Cómo ha aprendido lo que Usted sabe de la naturaleza de este lugar? ***Mencionar cada opción y preguntar sí o no***

| Se ha capacitado (charlas, talleres) |  |
| --- | --- |
| Lee artículos o revistas |  |
| Ha visto programas de televisión |  |
| Ha aprendido solo a través de su experiencia directa con la naturaleza |  |
| Tradiciones que se traspasan de generación en generación |  |
| Conocimiento Universitario |  |
| Otras: |  |

¿Usted separa residuos, reutiliza o recicla?

| 1. Sí |  |
| --- | --- |
| 2. No |  |

***Preguntas sociodemográficas:***

| Edad (años) |  |
| --- | --- |
| Género | F:_____; M:_____ |
| Profesión y actividad/ocupación |  |

*Nivel de estudios*:

| Sin estudios |  |
| --- | --- |
| Enseñanza básica |  |
| Enseñanza media |  |
| Enseñanza Universitaria |  |
| Posgrado |  |

¿Qué otra(s) persona(s) considera Usted relevante(s) de contactar para responder esta entrevista? ***(preguntar por datos de contacto)***

¿Desea hacer alguna pregunta o dar algún comentario? ¿De qué manera se podría mejorar este instrumento?

**MUCHAS GRACIAS POR SU COLABORACIÓN**
