# Supplementary material for: Social preferences for ecosystem services in a biodiversity hotspot in South America
Source: PLoS One. 2019 Apr 22;14(4):e0215715. doi: 10.1371/journal.pone.0215715 (PMC6476511; doi:10.1371/journal.pone.0215715)
Supplement: S1 Table — Eigenvalues and variance explained by the analysis. Biplots were created using these data. (DOCX) [file pone.0215715.s002.docx]

**S1 Table. Resulting Factor Scores from RDA.** Eigenvalues and variance explained by the analysis. Biplots were created using these data.

|  | **F1** | **F2** | **F3** | **F4** | **F5** |
| --- | --- | --- | --- | --- | --- |
| Eigenvalue | 1.1848 | 0.6521 | 0.5248 | 0.3582 | 0.3321 |
| Variance explained | 32.3132 | 17.7860 | 14.3137 | 9.7684 | 9.0579 |
| Cumulative % | 32.3132 | 50.0992 | 64.4128 | 74.1813 | 83.2391 |
| **Ecosystem services** |  |  |  |  |  |
| Food from traditional agriculture | 0.8107 | 0.1215 | -0.1225 | 0.1268 | 0.2686 |
| Symbolic plants | 0.1845 | 0.2637 | 0.9502 | 0.0380 | 0.2586 |
| Drinking water | 0.6418 | 0.1419 | 0.3256 | -0.3149 | -0.5759 |
| Water for agriculture | 0.9353 | -0.2668 | -0.1897 | -0.0946 | 0.1714 |
| Conservation activities motivated by iconic threatened species | -0.1748 | 0.5939 | 0.2854 | -0.2653 | 0.2822 |
| Fresh air and climate change control | -0.5265 | -0.1262 | 0.3017 | 0.6479 | -0.2602 |
| Water regulation and retention | -0.6316 | 0.8070 | -0.3941 | -0.1554 | 0.0149 |
| Beekeeping | 0.4279 | 0.3556 | -0.1597 | 0.5196 | 0.2805 |
| Educational value | -0.6016 | -0.6751 | 0.1320 | -0.2576 | 0.4231 |
| **Stakeholder socio-demographic characteristics (occupation)** |  |  |  |  |  |
| Scientists | -0.2248 | 0.0846 | -0.1922 | -0.1017 | -0.0622 |
| Employees of the Chilean National Forest Corporation (CONAF) | -0.1428 | -0.0339 | -0.0815 | -0.3130 | 0.0066 |
| Enterprise managers/owners | 0.1284 | 0.2622 | 0.1959 | 0.0596 | 0.2710 |
| Educators in schools and colleges | -0.0376 | -0.2354 | 0.3878 | -0.0017 | -0.0309 |
| Employees of the local government | -0.1272 | -0.0867 | 0.0720 | 0.1731 | -0.0838 |
| NGO members | -0.3064 | -0.2345 | -0.0573 | 0.0999 | 0.0245 |
| Members of local organizations | 0.2588 | 0.0720 | 0.0063 | 0.0472 | -0.1835 |
| Small farmers | 0.4303 | 0.1052 | -0.2064 | 0.0977 | 0.0203 |
| Tourism workers | -0.1031 | 0.0746 | -0.0843 | -0.1435 | 0.0250 |
| **Other sociodemographic and cultural characteristics independent of occupation** |  |  |  |  |  |
| Knowledge about protection figures | -0.3401 | 0.0822 | 0.0846 | -0.1387 | 0.0842 |
| Rural | 0.3255 | -0.0801 | 0.0536 | 0.0641 | 0.0963 |
| Urban | -0.2469 | -0.0217 | 0.0404 | 0.0747 | -0.1136 |
| Environmental organization membership | -0.0044 | -0.0512 | 0.1283 | -0.0289 | -0.0467 |
| Protected areas visitor | -0.5553 | -0.0396 | 0.0307 | 0.0129 | 0.1122 |
| Recycling habits | -0.3425 | 0.1701 | 0.1351 | 0.1060 | 0.0434 |
