# Supplementary material for: Social preferences for ecosystem services in a biodiversity hotspot in South America
Source: PLoS One. 2019 Apr 22;14(4):e0215715. doi: 10.1371/journal.pone.0215715 (PMC6476511; doi:10.1371/journal.pone.0215715)
Supplement: S2 Table — Bold Variables did not Present Any of the Largest Square Cosine Values and were Excluded from RDA. (DOCX) [file pone.0215715.s003.docx]

**S2 Table.** Results from the PCA analysis. Bold variables did not present any of the largest square cosine values in the 5 components selected and were excluded from the RDA.

|  | F1 | F2 | F3 | F4 | F5 |
| --- | --- | --- | --- | --- | --- |
| Eigenvalue | 2.1498 | 1.4618 | 1.2942 | 1.2095 | 1.0083 |
| Variance explained | 19.5434 | 13.2892 | 11.7655 | 10.9958 | 9.1660 |
| Cumulative % | 19.5434 | 32.8326 | 44.5981 | 55.5938 | 64.7598 |
| Ecosystem services | | | | | |
| Food from agriculture | **0.3256** | 0.0743 | 0.1155 | 0.1072 | 0.0002 |
| **Medicinal plants** | 0.0622 | 0.1435 | 0.1723 | 0.1781 | 0.1574 |
| Symbolic plants | 0.0000 | 0.0438 | 0.0897 | **0.5986** | 0.0335 |
| Drinking water | **0.4589** | 0.0042 | 0.0725 | 0.0452 | 0.0661 |
| Water for agriculture | **0.3809** | 0.0172 | 0.0019 | 0.0132 | 0.1261 |
| Conservation activities carried out by different actors motivated by iconic threatened animal and plant species | 0.0162 | 0.0043 | **0.5157** | 0.0757 | 0.0175 |
| **Genetic pool of the plant communities in Central Chile** | 0.3490 | 0.1111 | 0.0014 | 0.0153 | 0.0068 |
| Fresh air and climate change control | 0.1424 | 0.0612 | 0.0935 | 0.0283 | **0.3612** |
| Water regulation and retention | 0.0346 | **0.5738** | 0.0381 | 0.1075 | 0.0064 |
| Beekeeping | 0.2087 | 0.0107 | 0.1450 | 0.0256 | **0.2320** |
| Educational value | 0.1712 | **0.4178** | 0.0484 | 0.0146 | 0.0010 |
| Stakeholders characteristics (occupation) | | | | | |
| Scientists | 0.1579 | **0.2145** | 0.0018 | 0.1275 | 0.0936 |
| Enterprise managers/owners | 0.0524 | 0.0049 | 0.1059 | 0.0923 | 0.0309 |
| Employees of the Chilean National Forest Corporation (CONAF) | 0.0824 | 0.0055 | 0.0899 | 0.0941 | **0.1754** |
| Members of local organizations | **0.3500** | 0.0204 | 0.0050 | 0.0523 | 0.0079 |
| NGO members | **0.3202** | 0.0541 | 0.0663 | 0.1224 | 0.1110 |
| Tourism workers | 0.0662 | 0.0028 | 0.2184 | 0.2201 | 0.0692 |
| Small farmers | **0.6572** | 0.0201 | 0.0537 | 0.0224 | 0.0260 |
| Employees of the local government | 0.1999 | 0.0072 | 0.0331 | 0.0840 | 0.0035 |
| Educators in school and colleges | 0.0168 | 0.1192 | 0.0003 | **0.4940** | 0.0632 |
| Other stakeholder sociodemographic and cultural characteristics independent of occupation | | | | | |
| Knowledge of protection figures | **0.2599** | 0.0159 | 0.1761 | 0.0011 | 0.0004 |
| **The biosphere reserve category represents improvements in the area** | 0.0447 | 0.2159 | 0.0023 | 0.0295 | 0.0002 |
| **Previously consulted about topics related to the biosphere reserve** | 0.1114 | 0.0533 | 0.1843 | 0.0069 | 0.0213 |
| Rural | **0.4287** | 0.1128 | 0.0500 | 0.0337 | 0.0082 |
| Urban | 0.3304 | 0.0506 | 0.0001 | 0.0052 | 0.1013 |
| Rural and urban^[[1]](#footnote-1)^ | 0.1145 | 0.0886 | 0.1966 | **0.2577** | 0.1418 |
| Environmental organization membership | 0.0127 | **0.3172** | 0.2174 | 0.0002 | 0.1574 |
| Protected areas visitor | **0.8337** | 0.0172 | 0.0058 | 0.0035 | 0.0000 |
| Learned about nature through formal environmental education | 0.1968 | 0.0006 | 0.0644 | 0.0892 | 0.0143 |
| Learned about nature through environmental news and magazines | 0.1980 | 0.1016 | 0.0664 | 0.0182 | 0.0001 |
| Learned about nature through environmental television programs | 0.0715 | 0.1221 | **0.4289** | 0.0000 | 0.0086 |
| Learned about nature through direct experience | 0.1437 | **0.2255** | 0.0009 | 0.0648 | 0.0780 |
| Learned about nature through familiar traditions | 0.1016 | 0.0079 | 0.0783 | 0.0238 | 0.0134 |
| University knowledge | **0.3545** | 0.3507 | 0.0263 | 0.1120 | 0.0129 |
| **Other sources of knowledge** | 0.0212 | 0.1147 | 0.0615 | 0.0148 | 0.0115 |
| Recycling habits | 0.2828 | 0.0146 | 0.0207 | 0.0136 | **0.2941** |
| **Gender-Male** | 0.0129 | 0.0931 | 0.1413 | 0.1175 | 0.0357 |
| **Gender-Female** | 0.0129 | 0.0931 | 0.1413 | 0.1175 | 0.0357 |
| University level | 0.0291 | 0.0432 | 0.2559 | 0.1647 | 0.0039 |
| Postgraduate level | **0.7397** | 0.0461 | 0.0057 | 0.0004 | 0.0103 |
| Secondary school | 0.2471 | 0.0689 | 0.1707 | 0.0583 | 0.0113 |
| Primary school | 0.3003 | 0.0135 | 0.0197 | 0.0033 | 0.0066 |
| **Age** | 0.0372 | 0.1770 | 0.0074 | 0.0691 | 0.0064 |

1. People who feel neither rural nor urban. [↑](#footnote-ref-1)
